# Comprehensive Review of Red Meat Consumption and the Risk of Cancer

**DOI:** 10.7759/cureus.45324

**Published:** 2023-09-15

**Authors:** Barath Prashanth Sivasubramanian, Mihir Dave, Viraj Panchal, Johnnie Saifa-Bonsu, Srujana Konka, Farahnaz Noei, Sanchitha Nagaraj, Umabalan Terpari, Priya Savani, Pratikkumar H Vekaria, Vikramaditya Samala Venkata, Lokesh Manjani

**Affiliations:** 1 Infectious Diseases, University of Texas Health Science Center at San Antonio, San Antonio, USA; 2 Internal Medicine, Employees State Insurance Corporation Medical College (ESIC-MC) & Post Graduate Institute of Medical Sciences and Research (PGIMSR), Chennai, IND; 3 Internal Medicine, Medical College Baroda, Vadodara, IND; 4 Internal Medicine, Smt. Nathiba Hargovandas Lakhmichand Municipal Medical College, Ahmedabad, IND; 5 Internal Medicine, University of Ghana Medical School, Accra, GHA; 6 Internal Medicine, Prathima Institute of Medical Sciences, Nagunur, IND; 7 Internal Medicine, University of Ottawa, Ottawa, CAN; 8 Internal Medicine, Ramaiah Medical College, Bengaluru, IND; 9 Internal Medicine, Hospital Seri Manjung, Manjung, MYS; 10 Internal Medicine, Surat Municipal Institute of Medical Education & Research, Surat, IND; 11 Internal Medicine, Prisma Health University Medical Group, Greenville, USA; 12 Internal Medicine, Cheshire Medical Center, Keene, USA; 13 Internal Medicine, MedStar Washington Hospital Center, Washington, USA

**Keywords:** colon cancer, pancreatic cancer, breast cancer, gastrointestinal research, red meat

## Abstract

Red and processed meat consumption rates are increasing in the United States. In this review, we present the current evidence that links red meat consumption and cancer development. A literature search was conducted in the PubMed and Google Scholar databases to review red meat consumption and its association with breast cancer and gastrointestinal cancer. Due to the presence of heme iron, which triggers oxidative reactions that eventually result in tumor formation, red meat consumption is strongly associated with the development of breast cancer. Ingestion of red meat increases Helicobacter pylori infections, resulting in enhanced expression of the CagA gene and the secretion of pro-inflammatory cytokines. This is the leading cause of gastric cancer. There is a strong correlation between heterocyclic amines and polycyclic aromatic hydrocarbons in red meat and the development of pancreatic cancer. However, additional research is necessary to confirm this finding. Adult colorectal cancer is caused by the formation of heterocyclic amines and DNA adducts due to the intake of red and processed meats cooked at higher temperatures. The consumption of poultry is associated with a reduced risk of breast and gastrointestinal cancers, but the results are inconsistent. The evidence is strong for the association between red meat and breast cancer and most gastric cancers. The presence of aromatic hydrocarbons, heterocyclic amines, and heme iron in red meat has been found to be behind tumorigenesis. Poultry has been shown to have a low association with cancer, but additional research is needed.

## Introduction and background

In many parts of the world, people prioritize eating meat as a primary food group. Meat provides a significant source of protein and fat, as well as essential vitamins and nutrients like iron (Fe), zinc (Zn), vitamin A, and vitamin B [1]. Red (e.g., beef, lamb, goat) and processed meats (e.g., hot dogs, beef jerky, sausage) make up the majority of total meat consumption and have been rising in the United States [2]. Consuming processed meat has been linked to a 6% higher risk of breast cancer [mainly in postmenopausal women], an 18% higher risk of colorectal cancer, a 21% higher risk of colon cancer, and a 22% higher risk of rectal cancer [3,4]. Red and processed meats are considered a hidden risk factor for stomach cancer [5]. According to the World Cancer Research Fund 2012 report on pancreatic cancer, the evidence regarding the contribution of red meat and processed meat to an elevated risk was deemed to be "limited" due to the contradictory evidence [6].

Exogenous hormones used in beef cattle may leave behind hormone residues that increase the risk of developing estrogen receptor+ (ER+) tumors in breast cancer [4]. High-temperature cooking of red meat can create carcinogenic heterocyclic amines, which may influence the pathophysiology of breast cancer [4]. Heterocyclic amines and polycyclic aromatic hydrocarbons are major contributors to breast, stomach, pancreatic, and colorectal cancer [3,5,6]. The amount of red meat consumed per day and the heme iron from red meat are thought to be risk factors for developing stomach cancer [5].

In the available literature, there is a wide range of results, which can be attributed to the studies' diverse inclusion criteria. The quality of the evidence linking red and processed meat to negative health consequences is still uncertain. Concerning breast cancer, there is little evidence linking red meat consumption to the presence of tumor hormone receptors [4]. The associations between red and processed meat consumption and pancreatic cancer risk remain unclear [6]. Studies on particular red meats like beef and pork and their association with colon cancer are also limited [7]. Further research is needed to evaluate the effect of red or processed meat consumption on specific histological subtypes of esophageal cancer [8]. Several studies suggest varied results regarding the associations between red meat and gastric cancer [9].

## Review

Methods

A literature search on secondary data was conducted using PubMed and Google Scholar databases to better understand the relationship between red meat and the risk of cancer. English-language peer-reviewed publications on red meat consumption by humans were included here. The terms used were relevant keywords such as "red meat," "breast cancer," "stomach cancer," and "pancreatic cancer." After removing duplicates, studies that did not fit the criteria were excluded. We reviewed the entire document, and the references to the included articles were also screened.

Results and discussion

Breast Cancer

Several mechanisms have been proposed to explain the association between red and processed meat consumption and breast cancer risk. These include carcinogenic byproducts formed during high-temperature cooking of red meat, the presence of fat, heme iron, and the animal sugar molecule N-glycolylneuraminic acid, which may promote inflammation, oxidative stress, and tumor formation, and in some countries, the presence of hormone residues from exogenous hormones used to stimulate the growth of beef cattle [10]. Table 1 depicts the literature review describing red meat and cancer risk. 

**Table 1:** Literature review describing red meat and cancer risk

S no	Study name	Objectives of the study	Study finding
	Breast cancer		
1.	Anderson et al; 2018 [11]	Red and processed meat intake may be risk factors for breast cancer.	In postmenopausal women, processed meat intake, but not red meat may increase the risk of breast cancer.
2.	Diallo et al; 2017 [12]	Relationships between red and processed meat consumption and the risk of breast cancer.	Red meat intake was associated with increased breast cancer risk.
3.	Farvid 2018 [4]	The relation between red meat and processed meat consumption with breast cancer incidence.	High processed meat consumption was found to be associated with increased breast cancer.
4.	Farvid et al 2021 [3]	Associations between the consumption of red meat and processed meat with the incidence of various malignancies.	High red meat and processed meat intake were positively associated with the risk of breast cancer and various other malignancies.
5.	Kazemi et al 2021 [13]	The associations between red meat and processed meat with risk of breast cancer.	Low intakes of red and processed meat were associated with lower risks of breast cancer.
6.	Poorolajal et al; 2021 [14]	Identifying factors on the risk of breast cancer incidence.	Red meat was not significantly associated with the risk of breast cancer incidence.
7.	Zeraatkar et al; 2019 [15]	Red meat intake on clinically important outcomes.	Red meat restriction may have minimal or no effect on cancer incidence.
8.	Wu et al; 2016 [10]	Association between dietary protein sources and the risk of breast cancer.	High red meat and processed meat intake may be risk factors for breast cancer.
9.	Lo et al; 2020 [16]	Association between consumption of different types of meat, meat mutagens, and incident invasive breast cancer.	Consumption of red meat may increase the risk of invasive breast cancer. The consumption of poultry may be associated with a decreased risk.
10.	Farvid et al; 2015 [17]	Consumption of red meat and other protein sources in relation to breast cancer risk.	Higher consumption of red meat during adolescence was associated with premenopausal breast cancer. Substituting other protein sources in the adolescent diet may decrease premenopausal breast cancer risk.
11.	Chandran et al; 2013 [18]	Association between consuming meat and breast cancer risk.	The magnitude of the associations between breast cancer risk and consumption of red meat and poultry varied between African American and Caucasian women, with additional differences noted by menopausal status and hormone receptor status in Caucasian women.
	Gastric cancer		
S no	Study name	Objectives of the study	Study finding
1.	Zhao et al; 2017 [9]	Associations between red and processed meat consumption and risk of gastric cancer.	Cohort studies found no association between red and processed meat consumption and the risk of gastric cancer, whereas case-control studies found positive associations.
2.	Kim et al; 2019 [5]	Associations between red, processed, and white meat with gastric cancer.	Increased consumption of white meat may reduce the risk of gastric cancer, whereas red or processed meat may increase the risk.
3.	Song et al; 2014 [19]	Association between red meat consumption and stomach cancer risk.	Red meat consumption could pose a risk factor for stomach cancer.
4.	Ferro et al; 2020 [20]	Determine the link between meat consumption and the risk of gastric cancer.	Adherence to dietary recommendations to reduce meat consumption could contribute to a decrease in the occurrence of gastric cancer.
5.	Wilunda et al; 2022 [21]	Association of meat consumption with gastric cancer risk.	Meat consumption was not associated with gastric cancer risk.
6.	Collatuzzo et al; 2022 [22]	Identifying the association of different meat types with esophageal and gastric cancer.	Red meat intake is associated with gastric cancer, but not esophageal cancer.
7.	Zhu et al; 2013 [23]	Red and processed meat as a risk factor for gastric cancer.	Consumption of red and processed meat contributes to increased gastric cancer risk.
8.	Maddineni et al; 2022 [24]	Association between diet and gastric cancer risk.	Strong evidence that animal products (meats, eggs, and dairy) increase the risk of gastric cancer.
9.	Vahid and Davoodi et al; 2021 [25]	Nutritional risk factors for gastric cancer.	Red meat increases the risk of gastric cancer.
10.	Zamani et al; 2013 [26]	Relation between meat consumption and the risk of developing gastric cancer.	Red meat intake is positively associated with gastric cancer.
11.	Bonequi et al; 2013 [27]	Identifying risk factors for gastric cancer.	Consistent with multifactorial pathogenesis, smoking, alcohol use, high red meat or processed meat consumption, excess salt intake, and carriage of IL1RN*2 were each associated with a modest increase in gastric cancer risk.
	Pancreatic cancer		
S no	Study name	Objectives of the study	Study finding
1.	McCoullough et al; 2018 [28]	Association of meat consumption with pancreatic cancer risk.	The associations between meat consumption and the risk of pancreatic cancer remain unknown.
2.	Pericleous et al; 2014 [29]	Evaluating the role of dietary components in pancreatic cancer.	Avoid red meat cooked at high temperatures and opt instead for poultry or fish. Total fat must be decreased.
3.	Ruan et al; 2019 [30]	Association between red meat and processed meat and risk of colorectal, stomach, pancreatic cancer, and esophageal cancer.	Consuming red and processed meat is associated with a small but substantial cancer risk.
4.	Beaney et al; 2017 [31]	Risk of involvement of carcinogens in red meat in causing pancreatic cancer.	Red and processed meats may contribute to the development of pancreatic cancer.
5.	Huang et al; 2019 [32]	Red meat intake and pancreatic carcinogenesis.	Red meat intake was non significantly associated with pancreatic cancer.
6.	Ghorbani et al; 2015 [33]	Compare the frequency intake of different food items and their cooking methods and the risk of pancreatic cancer.	Increased frequency of intake of bread, rice, red meat, and deep-fried vegetables can increase pancreatic cancer risk.
	Colon cancer		
S no	Study name	Objectives of the study	Study finding
1.	Mehta et al; 2020 [34]	Associations between cooking practices and the risk of colorectal cancer.	High consumption of processed meats and grilled/barbecued red meat products was associated with an increased risk of colorectal cancer.
2.	Helmus et al; 2013 [35]	Assessing the formation of mutagenic heterocyclic amines and polycyclic aromatic hydrocarbons to drive the association of colon cancer with meat consumption.	Adds evidence supporting the role of heterocyclic amines and polycyclic aromatic hydrocarbons derived from red meat, but not white meat, in the environmental carcinogenesis of colon cancer.
3.	Ananthakrishnan et al; 2015 [36]	Interaction between red meat intake and colorectal cancer.	High red meat intake was associated with an increased risk of colorectal cancer.
4.	Bernstein et al; 2015 [37]	Association between red meat consumption and colorectal cancer.	The consumption of processed meat was associated with an increased risk of colorectal cancer, particularly distal cancer.
5.	Egeberg et al; 2013 [38]	Effects of specific red meat subtypes on colon cancer or rectal cancer risk.	The risk of colon cancer was significantly increased by consuming more lamb, while the risk of rectal cancer was increased by consuming more pork. Fish consumption was associated with a significantly lower risk of colon cancer, but not rectal cancer. The substitution of white meat for red meat had no effect on either risk.

For the relation between red meat consumption and developing breast cancer, there are studies conducted in a large prospective cohort that have shown that red meat intake is significantly associated with an overall increase in cancers and also increases the risk of developing breast cancer [12]. Even with red meat, there are direct associations with developing breast cancer shown in two meta-analyses, in which both have demonstrated a positive association of increased breast cancer risk with red meat as well as processed meat [10,39]. While Anderson et al. report that consumption of processed meat may be linked to an increased risk of breast cancer compared to red meat, and this association was significant in postmenopausal females compared to premenopausal [11], similar reports were seen by Wolk et al. and Inoue-Choi et al. [40,41]. Positive associations were found between red meat intake and the risk of regional or distant cancer and between processed meat intake and localized cancer. This is likely due to the observed positive association between nitrite intake from processed red meat and the risk of localized cancer and the positive association between heme iron intake and breast cancer, regardless of cancer stage [41]. Farvid et al. showed that unprocessed red meat intake was associated with a 6% higher risk of breast cancer (RR 1.06, 95% CI=0.99-1.14), and consumption of processed meat was associated with a 9% higher risk (RR 1.09, 95% CI=1.03-1.16) [4]. Boldo et al. say that by moderately consuming well-done, stewed red meat or even pan-fried or bread-coated fried white meat, the risk of developing breast cancer can be reduced [42].

Lo et al. pointed out that by substituting poultry for red meat, the risk of invasive breast cancer can also be reduced [16]. Lo et al. explain that this may be due to residual confounding, as those who reported higher consumption of poultry had healthier dietary patterns than those with lower consumption. Furthermore, the inverse association with poultry may be due to differences between red meat and poultry, such as saturated fat content or heme iron. Additionally, poultry consumption, compared to red meat consumption, may be associated with lower levels of mutagenic activity, oxidative stress, and DNA damage [16]. However, other studies show that consumption of red meat and poultry is also associated with breast cancer [18]. Further research should investigate potential mechanisms that may explain the protective effect of poultry consumption on the risk of breast cancer.

Cicco et al. emphasized the evidence available that a healthy dietary pattern that consists of a high proportion of unrefined cereals, vegetables, nuts, fruits, and oil like olive oil, along with a moderate to low intake of saturated fatty acids and red meat, can improve overall survival in breast cancer survivors [43]. There is little certainty that suggests that diets that are restricted in red meat may not affect cancer mortality and incidence [15].

Gastric Cancer

Heme iron, which is abundantly contained in red meat, has been identified as a risk factor for gastric cancer [5]. This is due to its role in the endogenous formation of carcinogenic N-nitroso compounds (NOCs) and its ability to induce DNA damage and oxidative stress, which contribute to the formation of DNA adducts. Iron is also a critical factor for bacterial growth in H. pylori, which is a major risk factor for gastric cancer [5]. Collatuzzo et al. suggest that the potential mechanisms of carcinogenesis from red meat consumption may include the endogenous formation of genotoxic N-nitroso compounds (NOCs), heterocyclic amines, and polycyclic aromatic hydrocarbons produced through high-temperature cooking, as well as iron and agents associated with meat processing, which may all cause oxidative stress [22].

The majority of studies show a significant association between red meat consumption and the development of gastric cancer. Some studies have found that red meat consumption is linked to an increased risk of gastric cancer [5,19,20,23,26,27,44-48]. For example, Kim et al. found that both red and processed meat consumption were associated with a higher risk of gastric cancer, and Song et al. found that the highest level of red meat intake was associated with a 37% increased risk of gastric cancer [5,19]. A meta-analysis by Bonequi et al. showed a positive association between red meat and the carriage of IL1RN*2 each associated with a moderate increase in gastric cancer risk [27]. Epplein et al. showed that individuals with Helicobacter pylori infections (seropositivity to 5-6 virulent H. pylori proteins) and an increasing intake of red meat were at increased risk of gastric cancer. This suggests that in gastric cancer etiology, the increase in expression of H. pylori CagA, secretion of pro-inflammatory cytokines, and rate of cell replication could be the reason. Evidence of this H. pylori is seen in the analysis of 36 H. pylori strains from individuals in two regions in Colombia with differing incidences of gastric cancer, which found that increased salt concentrations affect CagA expression differently in different strains [45]. Collatuzzo et al. found that higher consumption of total, red, and processed meat was significantly associated with an increased risk of esophageal cancer and non-cardia gastric cancer, but not cardia gastric cancer [22]. Additionally, meat intake has been linked to the promotion of the growth of Helicobacter, which is the main risk factor for non-cardia gastric cancer [22]. However, other studies, such as the systematic review and meta-analysis by Zhao et al. and the cohort study by Wilunda et al., have found mixed or no associations between red meat consumption and gastric cancer risk [9,21]. It is important to note that Zhao et al. found that while case-control studies yielded positive associations between red and processed meat consumption and gastric cancer risk, cohort studies showed negative or null associations [9]. Wilunda et al. found that higher chicken consumption was associated with a reduced distal gastric cancer risk in women, but there was no association between meat consumption and total gastric cancer risk [21]. Zamani et al. found that individuals with the highest consumption of white meat had a statistically significant reduced risk of gastric cancer when compared to those with the lowest consumption of white meat [26].

Some studies showed a strong body of evidence that alcohol, processed foods, high salt intake, high fat intake, and foods with animal products (meats, eggs, and dairy) increase the risk of gastric cancer, but the Mediterranean diet, or diet with a large proportion of fresh fruits and vegetables and certain micronutrients, mainly selenium and vitamin C, is protective [24,25,45,49]. Modern nutrition recommends the Mediterranean diet (MD), characterized by high consumption of vegetables, fruits, cereals, beans, nuts, and olive oil; moderate consumption of fish, white meat, eggs, dairy products, and alcohol; and low consumption of red meat, processed meats, and sugary or fatty foods. Olive oil is the major source of lipids, and Escrich's research has suggested that extra virgin olive oil confers low tumor aggressiveness, likely due to molecular changes in tumors, such as in the composition of cell membranes, the activity of signaling proteins, and gene expression, which may lead to lower proliferation, higher apoptosis, and lower DNA damage [49-55]. Wie et al. showed that risk factors such as low intake of fruits and vegetables, high intake of red meat, and obesity are seen in gastric cancers [48]. Ferro et al. also found that adherence to dietary recommendations to reduce meat consumption may contribute to a reduction in the risk of gastric cancer [20].

While some studies have found a link between red meat consumption and gastric cancer risk, more research is needed to understand the relationship between the two. It is important to consider the findings of all relevant studies, including those that have found mixed or no associations.

Pancreatic Cancer

There is a strong correlation between pancreatic cancer and the presence of heterocyclic amines (HCAs) and polycyclic aromatic hydrocarbons (PAHs) in red meat. PAH, a carcinogen of type A, is primarily associated with smoking and cooking methods such as grilling and barbecuing [33]. A potential link between acrylamide in rice or bread and the pathogenesis of pancreatic cancer is suspected [33].

A systematic review of the literature on the relationship between red meat and pancreatic cancer has yielded mixed results. Some studies have found a positive association between red meat consumption and pancreatic cancer risk [29,56,57,31,58,33]. For example, Taunk et al. found that the risk of pancreatic cancer significantly increased with the intake of total meat, red meat, and high-temperature cooked meat, and Petrick et al. found that total red meat intake was associated with a 65% increased risk of pancreatic cancer, but the results were not significant [56,57]. Beaney et al. also found that red and processed meats may be involved in pancreatic carcinogenesis, but their significance could not be established [31]. Jansen et al. also suggested that certain unsaturated fatty acids may decrease pancreatic cancer risk, while well-done red meat or meat mutagens may increase it [58]. On the other hand, other studies have found no association between red meat consumption and pancreatic cancer risk [28,57,59,32]. The inconsistencies between diet and cancer may be due to variations in the underlying gene polymorphisms that regulate the metabolism of components of the diet or the antioxidant defense [58]. Huang et al. found that the incidence of pancreatic cancer was not associated with different ethnicities or red meat consumption [32].

Ghorbani et al. discovered that consuming more bread, rice, red meat (especially barbecued), and deep-fried vegetables was associated with an increased risk of pancreatic cancer, whereas consuming more fish was associated with a decreased risk of pancreatic cancer. [33]. The potential link between consuming rice or bread and the risk of pancreatic cancer (PC) might be caused by the high levels of acrylamide in these food items, which are hypothesized to be involved in the pathogenesis of PC. Additionally, the positive correlation between consuming red meat, particularly barbecued meat, and PC risk could be explained by the high amounts of heterocyclic amines (HCAs) and polycyclic aromatic hydrocarbons (PAHs) present in these food items [33]. Ruan et al. also reported that red meat and processed meat had been consistently associated with an increased risk of colorectal, stomach, pancreatic, and esophageal cancer (processed meat only) [30].

McCoullough et al. found an increased risk of pancreatic cancer with poultry consumption [28]. The medication roxarsone, which contained arsenic and was used to control intestinal diseases in poultry, was discontinued in 2011. Higher inorganic arsenic exposure was found in the urine of humans who consumed a lot of poultry compared to non-consumers. Additionally, an ecological study in Florida showed that those living near arsenic-contaminated water wells had double the risk of pancreatic cancer. Although inorganic arsenic is known to cause cancers of the lung, urinary bladder, and skin, neither a 2012 International Agency for Research on Cancer (IARC) monograph on arsenic nor a meta-analysis of occupational exposures identified arsenic as a potential cause of pancreatic cancer [28].

*Colorectal Cancer* 

There are some theories for the pathogenesis behind the associations between colorectal carcinoma and red meat. One of them is the possibility of gut microbiomes, which influence the relationship between colorectal cancer (CRC) and diet, and the role of red meat in modulating the progress of CRC [60]. There are certain assumptions that the link between red meat and CRC is due to cooking the food at a higher temperature, which eventually results in the production of heterocyclic amines [61]. Several enzymes help in mediating this process, the most important being the N-acetyltransferase 2 (NAT2) enzyme. NAT2 activates the heterocyclic amines and helps in forming DNA adducts, which eventually damage the DNA; thus, the role of NAT2 enzyme activity also determines the progression of CRC [36]. It is also known that those with a phenotype of rapid acetylation by NAT2 have been associated with higher levels of DNA adducts when compared to those with a slower acetylation phenotype [62]. However, data from a retrospective case-control study showed that increased consumption of red meat was associated with CRC, and the relationship is not modified by the enzyme activity of NAT2 [36]. Nucleotide excision repair (NER) pathways are also found to be associated with CRC risk and may interfere with the association between well-done red meat and CRC risk; however, studies have not shown this to be significant [63]. Heterocyclic amines (HCAs) and polycyclic aromatic hydrocarbons (PAHs) that are derived from red meat play a role in developing CRC [35].

The risk of developing colorectal cancer (CRC) is linked with various factors, and among those are dietary factors and the lifestyle of a person, which may contribute to an increased incidence of the disease. This is well supported by an epidemiological study showing that increased amounts of red meat consumption are associated with an increased risk of CRC [64]. CRC risk is influenced by both the total intake of red meat and the frequency of intake [65]. Higher consumption of red and processed meat is associated with a higher risk of CRC [66]. A study of two large cohorts of men and women by Bernstein et al. [37], in which they repeatedly measured the dietary intake over two successive years, showed an association between developing distal colon cancer and higher consumption of processed meat. The same was inversely associated with unprocessed red meat consumption and distal colon cancer in the study [37]. It is also noted that increased consumption of barbecued or grilled red meat and processed meat is associated with an increased risk of CRC in women [34].

## Conclusions

The evidence is strong for the association between red meat and breast cancer and most gastric cancers. The presence of aromatic hydrocarbons, heterocyclic amines, and heme iron in red meat has been found to be behind tumorigenesis. Poultry has been shown to have a low association with cancer, but additional research is needed.
